# Dengue Virus Neutralizing Antibody Levels Associated with Protection from Infection in Thai Cluster Studies

**DOI:** 10.1371/journal.pntd.0003230

**Published:** 2014-10-16

**Authors:** Darunee Buddhari, Jared Aldstadt, Timothy P. Endy, Anon Srikiatkhachorn, Butsaya Thaisomboonsuk, Chonticha Klungthong, Ananda Nisalak, Benjawan Khuntirat, Richard G. Jarman, Stefan Fernandez, Stephen J. Thomas, Thomas W. Scott, Alan L. Rothman, In-Kyu Yoon

**Affiliations:** 1 Department of Virology, Armed Forces Research Institute of Medical Sciences, Bangkok, Thailand; 2 Department of Geography, University at Buffalo, Buffalo, New York, United States of America; 3 Department of Infectious Diseases, State University of New York at Syracuse, Syracuse, New York, United States of America; 4 Division of Infectious Diseases and Immunology, Department of Medicine, University of Massachusetts Medical School, Worcester, Massachusetts, United States of America; 5 Viral Diseases Branch, Walter Reed Army Institute of Research, Silver Spring, Maryland, United States of America; 6 Department of Entomology and Nematology, University of California at Davis, Davis, California, United States of America; 7 Fogarty International Center, National Institutes of Health, Bethesda, Maryland, United States of America; 8 Institute for Immunology and Informatics, University of Rhode Island, Providence, Rhode Island, United States of America; Pediatric Dengue Vaccine Initiative, United States of America

## Abstract

**Background:**

Long-term homologous and temporary heterologous protection from dengue virus (DENV) infection may be mediated by neutralizing antibodies. However, neutralizing antibody titers (NTs) have not been clearly associated with protection from infection.

**Methodology/Principal Findings:**

Data from two geographic cluster studies conducted in Kamphaeng Phet, Thailand were used for this analysis. In the first study (2004–2007), cluster investigations of 100-meter radius were triggered by DENV-infected index cases from a concurrent prospective cohort. Subjects between 6 months and 15 years old were evaluated for DENV infection at days 0 and 15 by DENV PCR and IgM ELISA. In the second study (2009–2012), clusters of 200-meter radius were triggered by DENV-infected index cases admitted to the provincial hospital. Subjects of any age ≥6 months were evaluated for DENV infection at days 0 and 14. In both studies, subjects who were DENV PCR positive at day 14/15 were considered to have been “susceptible” on day 0. Comparison subjects from houses in which someone had documented DENV infection, but the subject remained DENV negative at days 0 and 14/15, were considered “non-susceptible.” Day 0 samples were presumed to be from just before virus exposure, and underwent plaque reduction neutralization testing (PRNT). Seventeen “susceptible” (six DENV-1, five DENV-2, and six DENV-4), and 32 “non-susceptible” (13 exposed to DENV-1, 10 DENV-2, and 9 DENV-4) subjects were evaluated. Comparing subjects exposed to the same serotype, receiver operating characteristic (ROC) curves identified homotypic PRNT titers of 11, 323 and 16 for DENV-1, -2 and -4, respectively, to differentiate “susceptible” from “non-susceptible” subjects.

**Conclusions/Significance:**

PRNT titers were associated with protection from infection by DENV-1, -2 and -4. Protective NTs appeared to be serotype-dependent and may be higher for DENV-2 than other serotypes. These findings are relevant for both dengue epidemiology studies and vaccine development efforts.

## Author Summary

Dengue is caused by four different dengue virus serotypes (DENV-1, -2, -3, -4). Infection induces long-term protection against the same serotype, but only short-term protection, and possible enhancement, from different serotypes. DENV neutralizing antibody titers (NTs) are thought to mediate protection or modify disease. Association of NTs with protection from infection has not, however, been clearly demonstrated. We analyzed data from two geographic clusters studies conducted in Kamphaeng Phet, Thailand, in which DENV NTs just before virus exposure were compared between DENV-infected “susceptible” and non-infected “non-susceptible” subjects. NTs appeared to be associated with protection against DENV-1, -2, and -4, but at different NT cutoff levels, with the cutoff for DENV-2 appearing to be the highest. These findings are relevant for ongoing efforts to investigate dengue epidemiology and develop dengue vaccine candidates.

## Introduction

Dengue is caused by four closely related, but antigenically distinct dengue virus serotypes (DENV-1, -2, -3, -4) from the genus *Flavivirus* in the family *Flaviviridae* [1], [2]. In recent decades, dengue has expanded in tropical and subtropical regions and become one of the most prevalent vector-borne diseases of humans with approximately 2.5 billion people living with risk of infection. The annual global burden of dengue has been estimated to be 390 million infections with 96 million symptomatic cases [3]. Recently, the first human efficacy trial of a dengue vaccine candidate was completed in Thailand showing good neutralizing antibody response to all four DENV serotypes after vaccination, but no clinical efficacy against DENV-2 infection [4]. The relevance of DENV neutralizing antibodies for protection or modulation of DENV infection, therefore, remains unclear.

A primary infection with one serotype is thought to produce long-term protective immunity to re-infection with the homologous serotype. After a limited period of cross-protection, individuals who have had a primary DENV infection are susceptible to infection and disease by heterologous serotypes [5], [6]. In human challenge studies conducted by Sabin [7] in which DENV naïve individuals were infected with DENV-1 or DENV-2 and re-challenged with homologous or heterologous virus at different times after the initial challenge, protection against disease was observed for at least 18 months against the homologous serotype and at least 2 months against the heterologous serotype. Sabin also noted that infection appeared to be milder if heterologous re-challenge was performed up to 9 months after initial infection suggesting a period of partial heterologous protection. Epidemiological studies of dengue in endemic countries are consistent with this pattern of susceptibility [8]. Mathematical modeling of 38 years of dengue cases admitted to a pediatric hospital in Bangkok, Thailand, was consistent with approximately two years of heterologous protection against disease [9]. Analyses of symptomatic and subclinical DENV infections from prospective cohorts in Thailand and Nicaragua suggested a similar duration of cross-protection against disease [10], [11].

Heterologous protection is thought to be at least partly mediated by temporary cross-protective neutralizing antibodies from earlier infections [12]. Some studies have shown an association between pre-existing neutralizing antibody titers (NTs) and subsequent disease severity under certain conditions [13], [14], [15]. Endy *et al.* found such a correlation between homotypic NTs and subsequent viremia levels and disease severity for DENV-3, but not for DENV-1 and DENV-2 in a Thai pediatric cohort [14]. In contrast, Sirivichayakul *et al.* found no relationship between homotypic NTs and subsequent infection by DENV-1 or DENV-4 [16]. Up to now, no epidemiological study in humans has been able to demonstrate an association between pre-existing NTs and protection from infection.

One limitation of earlier prospective cohort studies has been that they measured neutralizing antibodies up to one year prior to infection. Neutralizing antibodies (and especially cross-reactive antibodies) decrease substantially over time, however, and their kinetics can be quite variable depending on factors such as DENV serotype from previous and current infection, disease severity, host genetics and immunological status [17]. Because neutralizing antibody status just before virus exposure is likely the most relevant for protection from infection, we sought to test the hypothesis that neutralizing antibody titers immediately before exposure was associated with the probability of infection by utilizing data from geographic cluster studies in which high DENV transmission activity has been demonstrated [18]. We showed an association between homotypic NTs and the likelihood of subsequent infection with DENV-1, -2 and -4.

## Methods

### Ethics Statement

Data from two different geographic cluster studies were used in the current analysis. The first study (called “KPSII”) was approved by the Institutional Review Boards (IRBs) of the Thai Ministry of Public Health (MOPH), Walter Reed Army Institute of Research (WRAIR), University of Massachusetts Medical School (UMMS), University of California at Davis (UCD), and San Diego State University (SDSU). The second study (called “DEVOL”) was approved by the IRBs of the Thai MOPH, WRAIR, UCD, and the State University of New York (SUNY) Upstate Medical University. Written informed consent was obtained from adult subjects (age ≥18 years) or the parents/guardians of child subjects (age <18 years); assent was obtained from child subjects ≥7 and <18 years of age.

### KPSII Study

In the current study, we used data from a prospective longitudinal cohort and geographic cluster study conducted from 2004 to 2007 among children living in Muang district, Kamphaeng Phet province (KPP) in north-central Thailand. The study methodology has been described previously [18], [19], [20]. Briefly, geographic cluster investigations were initiated by “index” cases from a longitudinal cohort of approximately 2,000 primary school children. Active school absence-based surveillance was used to detect symptomatic DENV infections in the cohort from June to November of each study year. Cohort children who were DENV positive by hemi-nested reverse transcriptase polymerase chain reaction (PCR) [21], [22] from an acute serum sample drawn within three days of illness onset served as an index case to initiate a positive cluster investigation around the index case house. Cohort children who were DENV PCR negative from an acute illness sample served as an index case for a negative cluster investigation. In each geographic cluster, ten to 25 contact subjects aged six months to 15 years living within 100 meters of the index case were enrolled regardless of clinical status. Contact subjects were evaluated at days 0 (i.e., same day as cluster initiation), 5, 10, and 15 by temperature measurement and symptom questionnaire. Serum samples were collected on days 0 and 15. Paired day 0 and 15 samples underwent DENV nested PCR and an in-house DENV/Japanese encephalitis virus (JEV) IgM capture enzyme-linked immunosorbent assay (ELISA) [23].

### DEVOL Study

We also used data from a geographic cluster study conducted from 2009 to 2012 in the same district of KPP, Thailand. The study methodology is being submitted in a separate manuscript. In the DEVOL study, geographic cluster investigations were initiated by “index” cases admitted to KPP hospital who were DENV positive by nested PCR [21], [22]. In each geographic cluster, adults and children ≥6 months of age living within 200 meters of the index case were enrolled regardless of clinical status. The distance was increased compared to the KPSII study to attempt to capture more DENV infections. Only individuals from households where at least one member had a history of fever in the previous seven days were considered for enrollment. Contact subjects were evaluated at days 0 (i.e., same day as cluster initiation) and 14 by temperature measurement, symptom questionnaire, and blood collection. Day 0 samples were tested by DENV nested PCR; day 14 samples were tested using DENV Detect NS1 ELISA (InBios, Seattle, Washington, USA) and, if positive, confirmed by DENV nested PCR. Paired day 0 and 14 samples were tested by an in-house DENV/JEV IgM capture ELISA [23].

### Selection of “Susceptible” and “Non-susceptible” Subjects

In order to assess neutralizing antibody status just prior to virus exposure, we first identified contact subjects from the two geographic cluster studies who were DENV positive by nested PCR at day 15 (KPSII study) or day 14 (DEVOL study). In the DEVOL study, only clusters in which there was a DENV-2 index case were utilized so that we could obtain data about DENV-2 which had not been detected at day 15 in the earlier KPSII study. Day 14/15 PCR positive subjects were classified as “susceptible” to DENV infection since they had confirmed infection. In order to create a “non-susceptible” comparison group, we identified contact subjects from the two studies who lived in the same house as another subject who was DENV positive on day 0, but were themselves DENV negative by nested PCR and IgM ELISA on both days 0 and 15 (KPSII study), or by NS1 ELISA and IgM ELISA on both days 0 and 14 (DEVOL study). These DENV negative “non-susceptible” subjects were presumed to have a high likelihood of exposure to DENV during the cluster investigation, yet still did not become infected. In KPSII, contact subjects who met these criteria were selected to be in the “non-susceptible” group. In DEVOL, given the wider age range, each DENV-2 positive “susceptible” subject was matched with two “non-susceptible” subjects by age (+/− 5 years), village and study year. In both the KPSII and DEVOL studies, the exposure serotype for all “non-susceptible” subjects was presumed to be the same as the infecting serotype for the index case from the same cluster. In all subjects, the day 0 blood sample was presumed to reflect the neutralizing antibody status just prior to exposure or infection. The number of DENV-3 infections from both the KPSII and DEVOL studies was not sufficient to be evaluated.

### Plaque Reduction Neutralization Test (PRNT)

To determine neutralizing antibody status just prior to exposure for all “susceptible” and “non-susceptible” subjects, day 0 blood samples were tested by an in-house plaque reduction neutralization test (PRNT) using all four DENV serotypes and JEV as previously described [24], [25]. A monolayer of Macaca mulatta kidney cells (LLC-MK2) was infected with 30–50 plaque-forming units of DENV in the presence of four-fold serial dilutions of heat-inactivated sample on a 12-well plate. For each dilution, the number of virus plaques was counted and compared to the number of plaques in a control where no sample was added. Reference strains were as follows: DENV-1 (Thailand/16007/1964), DENV-2 (Thailand/16681/1984), DENV-3 (Philippines/16562/1964), DENV-4 (Indonesia/1036/1976) [KPSII samples], DENV-4 (Thailand/C0036/2006) [DEVOL samples], and JEV (SA-14-14 vaccine strain). In addition to the reference strains, day 0 samples from subjects from DENV-2 clusters (i.e., clusters where the index case was infected with DENV-2) underwent PRNT using DENV-2 strains from Thailand that were isolated at the AFRIMS laboratory over a period of several decades as follows: Asian-American 1982 (#D82-165), Asian I 1974 (#D74-066), Asian I 1984 (#D84-501), Asian I 1994 (#D94-035), Asian I 2004 (#KDS00305), and homologous virus. Homologous viruses were all Asian I genotype and were cultured either from the same blood sample or from a subject from the same cluster or village in the same year. This was done in order to evaluate possible differences in PRNT titers against different DENV-2 strains given the lack of vaccine efficacy against DENV-2 reported in a recent dengue vaccine trial [4]. PRNT data was expressed as the reciprocal of the dilution causing 50% plaque reduction (PRNT50) as extrapolated from probit regression. In this study, our use of the terms “homotypic” and “heterotypic” neutralizing antibodies refers to relationships between serotype neutralizing antibodies based solely on the serotype-specific PRNT results along with the presumptive serotype circulating within a geographic cluster.

### Statistical Analysis

Analyses were performed comparing all subjects combined (i.e., “susceptible” versus “non-susceptible” subjects), and by comparing subjects according to exposure serotype. When comparing by serotype, subjects with the same serotype came exclusively from KPSII or DEVOL, but not both. Logistic regression models were employed to test the hypothesis that pre-existing NTs were associated with the probability of being PCR positive or negative. For each test, the dependent variable was the infection status of the subject. For each evaluated DENV strain used for PRNT, a simple model with only the log of NT, and a model adjusted for age were estimated. The model fit and predictive power were assessed for each model using the Akaike Information Criterion (AIC) and the area under receiver operating characteristic (ROC) curve (AUC). The AIC provides a measure of how closely the models fit the observed data, while penalizing for additional model complexity [26]. Lower AIC values indicate better model fit. The ROC curve is used to evaluate the accuracy of a diagnostic measure [27]. The 95% confidence interval (CI) of AUC was computed using 10,000 stratified bootstrap replicates [28]. Contingency tables were created to illustrate the predictive ability of individual NT cutoff values. The observed odds ratios were calculated and conditional maximum likelihood estimates of the 95% CIs based on Fisher's exact test were estimated to show the uncertainty associated with the observed odds ratios. All analyses were performed using the R environment for statistical computing. ROC curves were created and AUC was calculated using the pROC package for R [29].

## Results

Of 1599 contact subjects from 50 positive and 53 negative geographic cluster investigations in the KPSII study [18], [30], 12 subjects were found to be DENV PCR positive at day 15 and, therefore, considered to be “susceptible”: six had DENV-1 and six had DENV-4. All 12 subjects were PCR negative at day 0, and DENV IgM ELISA negative at days 0 and 15. No data or blood was collected after day 15. Twenty-two subjects from KPSII were selected as “non-susceptible”: 13 from DENV-1 clusters and nine from DENV-4 clusters. The predominant circulating serotype during the first two years of the KPSII study was DENV-4 and the last two years was DENV-1 [19].

Of 740 contact subjects from 195 DENV-2 geographic cluster investigations in the DEVOL study, five subjects were found to be DENV-2 PCR positive at day 14 and, therefore, considered as “susceptible.” All five subjects were PCR negative at day 0, and DENV IgM ELISA negative at days 0 and 14. No data or blood was collected after day 14. Ten subjects were selected as “non-susceptible” from DENV-2 clusters. The predominant circulating serotype during all years of the DEVOL study was DENV-2 (unpublished data).

**Figure 1:**
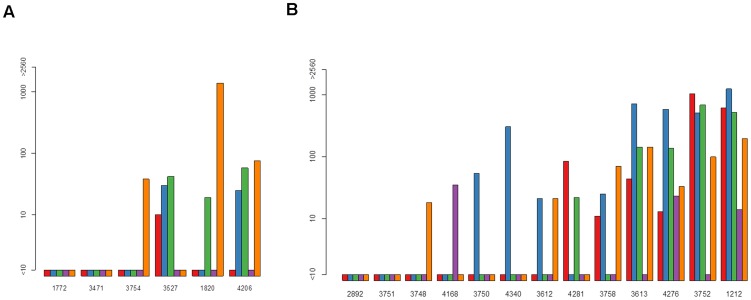
Pre-exposure neutralizing antibody titers (NTs) in dengue virus serotype 1 (DENV-1) “susceptible” and “non-susceptible subjects. Graphs show: (a) six DENV-1 PCR positive (“susceptible”) subjects; (b) 13 DENV-1 PCR negative (“non-susceptible”) subjects. NTs for each subject are against the reference strains for DENV-1, -2, -3, -4 and Japanese encephalitis virus (JEV) (red, blue, green, purple and orange, respectively).

**Figure 2:**
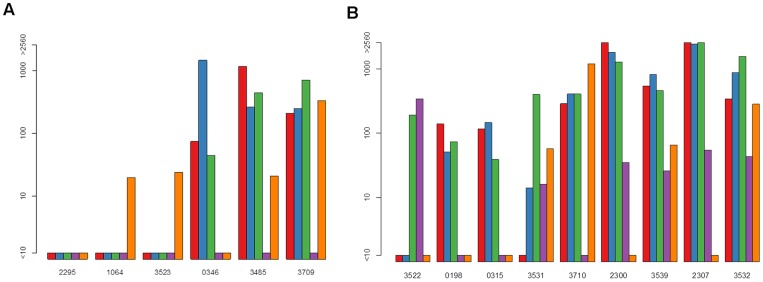
Pre-exposure neutralizing antibody titers (NTs) in dengue virus serotype 4 (DENV-4) “susceptible” and “non-susceptible” subjects. Graphs show: (a) six DENV-4 PCR positive (“susceptible”) subjects; (b) nine DENV-4 PCR negative (“non-susceptible”) subjects. NTs for each subject are against the reference strains for DENV-1, -2, -3, -4 and Japanese encephalitis virus (JEV) (red, blue, green, purple and orange, respectively).

**Figure 3:**
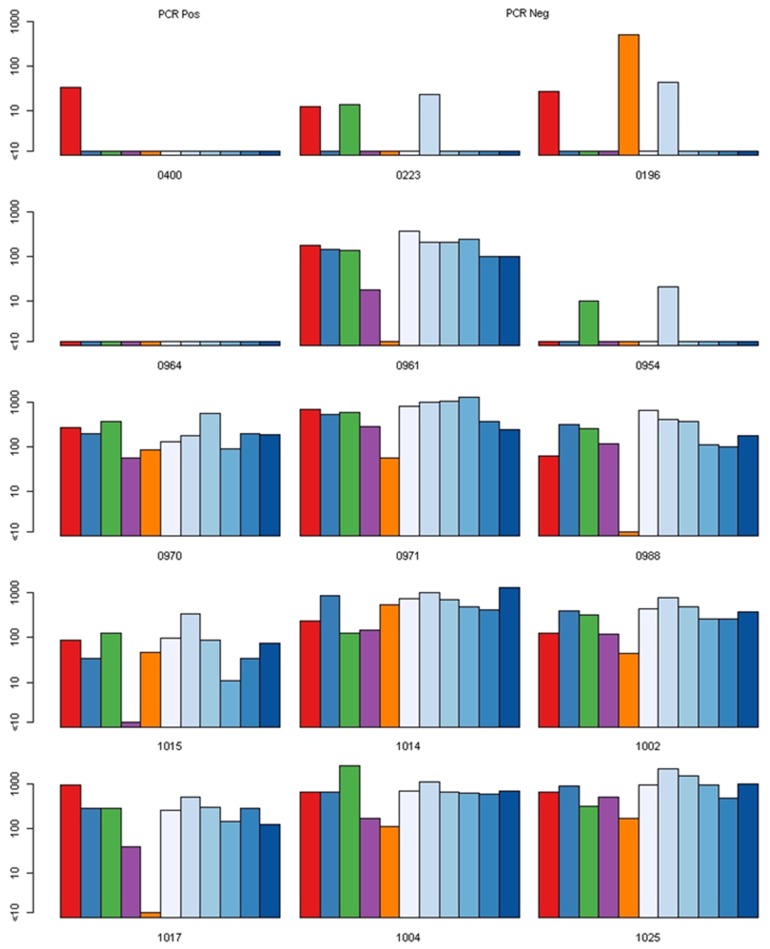
Pre-exposure neutralizing antibody titers (NTs) in dengue virus serotype 2 (DENV-2) “susceptible” and “non-susceptible” subjects. Each row shows one PCR positive (“susceptible”) subject and two matched PCR negative (“non-susceptible”) subjects. NTs for each subject are against (in order from left to right for each subject) the reference strains for DENV-1, -2, -3, -4 and Japanese encephalitis virus (JEV), and six different DENV-2 strains (Asian-American 1982 [#D82-165], Asian I 1974 [#D74-066], Asian I 1984 [#D84-501], Asian I 1994 [#D94-035], Asian I 2004 [#KDS00305]), and homologous DENV-2 Asian I strains).

Altogether, 49 subjects were available for analysis: 17 PCR positive “susceptible” subjects (six DENV-1, five DENV-2 and six DENV-4), and 32 PCR negative “non-susceptible” subjects (13 from DENV-1 clusters, 10 DENV-2 and 9 DENV-4). Table 1 lists characteristics of the 49 subjects. Figures 1, 2 and 3 show bar graphs of day 0 NTs for each subject. Seven “susceptible” compared with three “non-susceptible” subjects had DENV naïve NT profiles on day 0 (odds ratio = 6.460 [95% CI 1.198, 46.302]).

**Table 1:** Characteristics of dengue PCR positive (“susceptible”) and negative (“non-susceptible”) subjects.

	PCR positive (“Susceptible”)	PCR negative (“Non-susceptible”)
	DENV-1 clusters	
Median age, yr (range)	9.7 (2.5–11.6)	5.0 (1.3–12.6)
Female/Male, no.	3/3	8/5
PRNT profile		
DENV naïve, no.	3	3
DENV monotypic, no.	1	4
DENV multitypic, no.	2	6
	DENV-4 clusters	
Median age, yr (range)	7.7 (2.0–13.2)	8.3 (1.7–13.0)
Female/Male, no.	4/2	4/5
PRNT profile		
DENV naïve, no.	3	0
DENV monotypic, no.	0	0
DENV multitypic, no.	3	9
	DENV-2 clusters	
Median age, yr (range)	29.8 (1.4–55.7)	30.5 (0.9–61.4)
Female/Male, no.	2/3	9/1
PRNT profile		
DENV naïve, no.	1	0
DENV monotypic, no.	1	2
DENV multitypic, no.	3	8

PRNT to a serotype was considered positive if serotype-specific titer ≥10. PCR = polymerase chain reaction; DENV = dengue virus; PRNT = plaque reduction neutralization test; no significant differences are noted due to the small number of samples.

**Figure 4:**
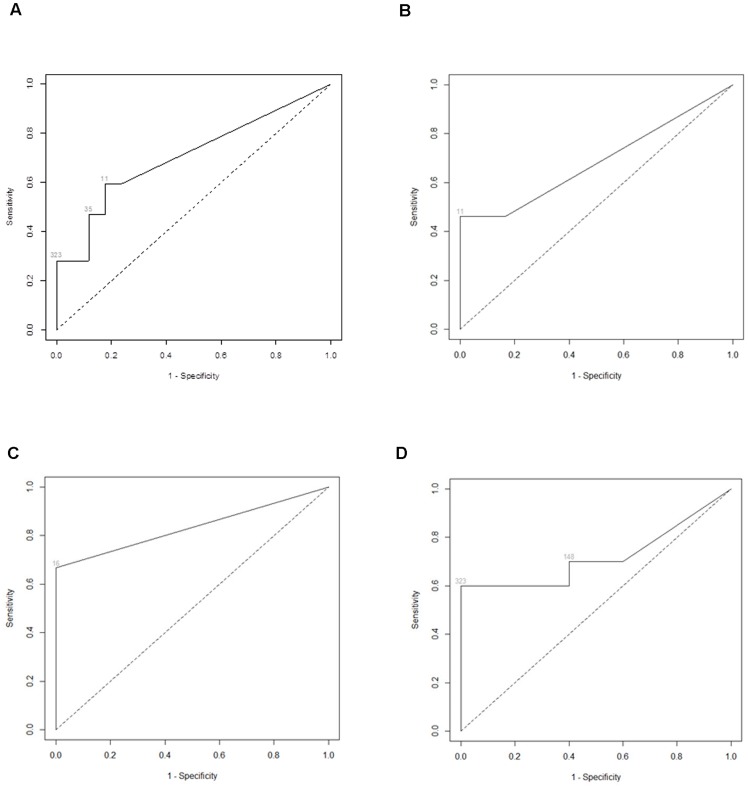
Receiver operating characteristic (ROC) curves for homotypic neutralizing antibody titers (NTs) against reference strains. Curves discriminate between dengue virus (DENV) PCR positive and negative subjects for: (a) all serotypes combined; (b) DENV-1; (c) DENV-4; (d) DENV-2.

When all serotypes were combined, PCR status was significantly associated with the log of homotypic NT both alone and adjusted for age (Table 2). ROC curves were created for all serotypes combined, and for each serotype separately (Figure 4). Considering subjects from just DENV-1 clusters, age-adjusted models to predict PCR status were better (based on AIC and AUC) when using homotypic DENV-1 NTs (AUC = 0.833 [95% CI 0.590, 1.000]) than heterotypic NTs (Table 3). For DENV-4 clusters, age-adjusted models were better using homotypic DENV-4 NTs (AUC = 0.889 [95% CI 0.667, 1.000]) than heterotypic NTs (Table 3). For DENV-2 clusters, age-adjusted models were better using both homotypic DENV-2 reference NTs (AUC = 0.740 [95% CI 0.460, 0.960]) and heterotypic DENV-4 reference NTs (AUC = 0.880 [95% CI 0.680, 1.000]) than other heterotypic NTs (Table 3). When different DENV-2 strains were used for PRNT, the AUC using Asian I 1974 strain appeared to have the best fit. The AUC using DENV-2 homologous virus was comparable to that using DENV-2 reference strain.

**Table 2:** Logistic regression analysis of the relationship between homotypic neutralizing antibody titers and dengue PCR status.^a^

Parameter	Estimate	Standard error	Z value	P value
	Model using log(homotypic NT) only			
Intercept	−0.011	0.382	−0.028	0.978
Log(homotypic reference strain NT)	−0.779	0.338	−2.303	0.021
	Model using log(homotypic NT) and age			
Intercept	−2.431	1.164	−2.088	0.037
Log(homotypic reference strain NT)	−1.549	0.524	−2.955	0.003
Log(age+1)	2.982	1.328	2.245	0.024

a Analysis combines all serotypes: 49 total subjects (17 PCR positive, 32 PCR negative).

PCR = polymerase chain reaction; NT = neutralizing antibody titer.

**Table 3:** Comparison of logistic regression models of neutralizing antibody titers to predict dengue PCR status.

PRNT strain	NT only		Age-adjusted model	
	AIC	AUC (95% CI)	AIC	AUC (95% CI)
	All serotypes combined^a^			
Homotypic reference	60.646	0.705 (0.570,0.828)	56.642	0.791 (0.659,0.906)
	DENV-1^b^			
DENV-1 reference	24.870	0.686 (0.487,0.846)	22.738	0.833 (0.590,1.000)
DENV-2 reference	24.813	0.699 (0.468,0.885)	25.636	0.782 (0.539,0.974)
DENV-3 reference	27.673	0.487 (0.269,0.731)	28.339	0.667 (0.372,0.923)
DENV-4 reference	25.170	0.615 (0.500,0.731)	25.188	0.782 (0.539,0.974)
JEV reference	27.614	0.474 (0.192,0.756)	28.561	0.667 (0.385,0.923)
	DENV-4^c^			
DENV-1 reference	22.540	0.704 (0.417,0.944)	24.398	0.704 (0.389,0.982)
DENV-2 reference	22.316	0.694 (0.389,0.944)	23.913	0.722 (0.407,0.944)
DENV-3 reference	19.625	0.722 (0.407,0.982)	21.639	0.722 (0.389,0.982)
DENV-4 reference	15.457	0.833 (0.667,1.000)	15.954	0.889 (0.667,1.000)
JEV reference	24.172	0.519 (0.232,0.815)	26.064	0.537 (0.241,0.852)
	DENV-2^d^			
DENV-1 reference	22.976	0.510 (0.180,0.840)	24.959	0.480 (0.140,0.840)
DENV-2 reference	22.270	0.720 (0.440,0.940)	22.708	0.740 (0.460,0.960)
DENV-3 reference	22.127	0.640 (0.310,0.920)	22.690	0.660 (0.340,0.920)
DENV-4 reference	20.949	0.750 (0.500,0.950)	20.759	0.880 (0.680,1.000)
JEV reference	22.087	0.660 (0.380,0.890)	23.957	0.660 (0.360,0.920)
DENV-2 Asian-Am 1982	22.195	0.760 (0.500,1.000)	22.291	0.780 (0.500,1.000)
DENV-2 Asian I 1974	20.196	0.760 (0.480,0.960)	17.863	0.880 (0.640,1.000)
DENV-2 Asian 1 1984	22.575	0.680 (0.400,0.920)	23.476	0.720 (0.420,0.940)
DENV-2 Asian I 1994	21.583	0.740 (0.480,0.940)	21.554	0.800 (0.520,0.980)
DENV-2 Asian I 2004	22.581	0.660 (0.370,0.900)	23.713	0.720 (0.420,0.960)
DENV-2 homologous	22.353	0.700 (0.420,0.930)	22.819	0.740 (0.440,0.960)

a 49 subjects (17 PCR positive, 32 PCR negative);

b 19 DENV-1 subjects (6 PCR positive, 13 PCR negative);

c 15 DENV-4 subjects (6 PCR positive, 9 PCR negative);

d 15 DENV-2 subjects (5 PCR positive, 10 PCR negative).

PCR = polymerase chain reaction; PRNT = plaque reduction neutralization test; NT = neutralizing antibody titer; AIC = Akaike Information Criterion; AUC = area under receiver operating characteristic curve; CI = confidence interval; DENV = dengue virus; JEV = Japanese encephalitis virus.

Models using neutralizing antibody titer alone and adjusted for age are shown.

Homotypic reference strain NT cutoff values as determined by ROC curves were used to create two-by-two contingency tables to demonstrate the relationship between individual NT cutoffs and PCR status (Table 4). Homotypic NT cutoff values were 11, 11, 16 and 323 for all serotypes combined, DENV-1, DENV-4, and DENV-2, respectively. The observed odds ratio for each sample is indicated and the 95% CI is presented to show the uncertainty associated with this measure. These CIs should not be used to assess statistical significance because they are post-hoc comparisons based on the cutoffs indicated by the ROC plot analysis. For all serotypes combined, the observed odds ratio of becoming infected (i.e., being PCR positive) if day 0 homotypic NT was ≥11 was 0.153 (95% CI 0.023, 0.700). For DENV-1 clusters, the observed odds ratio if homotypic NT was ≥11 was 0 (95% CI 0.000, 1.554). For DENV-4 clusters, the observed odds ratio if homotypic NT was ≥16 was 0 (95% CI 0.000, 0.842). For DENV-2, the odds ratio if homotypic NT was ≥323 was 0 (95% CI 0.000, 1.279).

**Table 4:** Contingency tables showing relationship between homotypic neutralizing antibody titer cutoff values and dengue PCR status.

NT cutoff status^a^	PCR negative, no.	PCR positive, no.	Observed odds ratio^b^ (95% CI)
	All serotypes combined		
Homotypic NT <11	13	14	0.153 (0.023,0.700)
Homotypic NT ≥11	19	3	
	DENV-1		
DENV-1 NT <11	7	6	0 (0.000,1.554)
DENV-1 NT ≥11	6	0	
	DENV-4		
DENV-4 NT <16	3	6	0 (0.000,0.842)
DENV-4 NT ≥16	6	0	
	DENV-2		
DENV-2 NT <323	4	5	0 (0.000,1.279)
DENV-2 NT ≥323	6	0	

a NTs from PRNT using reference strains;

b Observed odds ratio of becoming infected with given serotype if homotypic NT is ≥ cutoff value.

Conditional maximum likelihood estimates of the odds ratio are given to show the uncertainty associated with these values. Due to the post hoc examination of these cutoffs based on the ROC plot results, these intervals should not be used to assess statistical significance. PCR = polymerase chain reaction; NT = neutralizing antibody titer; CI = confidence interval; DENV = dengue virus.

## Discussion

Our analysis of subjects from geographic cluster studies indicates that pre-existing homotypic neutralizing antibody titers as measured by PRNT were positively associated with protection against infection by DENV-1, -2 and -4; a similar analysis could not be performed for DENV-3 because too few cases were available. Homotypic NTs were more strongly associated with protection than heterotypic NTs except in the case of DENV-2 infections, in which pre-existing heterotypic DENV-4 NTs were also positively associated with protection against DENV-2. This is the first human epidemiological study to report such an association and provides some support for the use of PRNT titers as a correlate of protective immunity in dengue epidemiology studies and vaccine development efforts.

Two major strengths of this study design likely explain why a significant association between NTs and protection from infection was observed in contrast to previous prospective cohort studies. Whereas earlier cohort studies typically analyzed NTs in blood samples collected up to six months or more prior to the incident DENV infection, our study provided blood samples from within two weeks prior to virus exposure, thus minimizing the confounding factor of variations in antibody kinetics. Although we could not document the exact day of virus inoculation in “susceptible” subjects, the two week interval was likely sufficient to ensure that day 0 blood collections occurred before exposure. This presumption is further supported by the fact that DENV IgM ELISA was negative at day 14/15 indicating that infection was still early in its course. Although we were able to confirm infection in “susceptible” subjects by a positive PCR, we could not prove exposure in PCR negative subjects. By requiring documentation of a DENV infection in the same house, however, our selection criteria for “non-susceptible” subjects maximized the likelihood of such exposure. Previous results from our cluster studies indicate a very high likelihood of virus exposure in houses with known DENV infection [18]. We have reported average DENV infection rates of greater than 30% over two weeks in houses with known DENV infection pointing to even higher rates of virus exposure if individuals who are exposed but immunologically protected are taken into account. These exposure rates are as high as is reasonably feasible in the natural setting (i.e., outside of an experimental challenge).

Whereas relatively low homotypic NT cutoff values were associated with protection against DENV-1 and DENV-4 in this study, much higher homotypic NT cutoffs were associated with protection against DENV-2. This indicates that protective NTs may depend, in part, on infecting serotype, with DENV-2 possibly requiring substantially higher PRNT titers. This finding needs to be tempered by the uncertainty in the mix of functional and non-functional antibody subpopulations that are being measured by PRNT, which could affect the generalizability of these NT cutoff values to other populations. Furthermore, although age was incorporated into the statistical analyses, the inclusion of adults in the DEVOL study compared to only children in the KPSII study could have introduced an additional confounding factor in interpreting NT values. Individual PRNT titers necessary for protection may, therefore, be difficult to define precisely. This difficulty could be compounded by the inherent variability in biological assays such as PRNT whether using similar or different methods (e.g., different cell lines) [25]. Nevertheless, our findings highlight the likelihood that traditionally accepted NT ranges for vaccine immunogenicity may not necessarily be relevant for protection from a natural DENV challenge. The lack of clinical efficacy against DENV-2 in the recent dengue vaccine trial in Thailand [4] may have partly been due to insufficiently high DENV-2 NTs. Moreover, vaccination in a primed population with the potential for interference from existing immunity makes this situation even more complex. What may be a protective NT level after natural DENV infection may not apply in the setting of multivalent vaccination, especially in DENV primed individuals.

Although our study indicates that PRNT titers may be related to protection from infection, these titers are likely to be surrogates for other immune responses that are more directly relevant to protection. For example, PRNT titers measured in Fc gamma receptor-bearing cells [31], [32] or after depletion of cross-reactive antibodies [33], and quaternary E-protein domain I/II hinge region antibody titers [34] have been proposed to be better indicators of protection. Conversely, E-protein domain II fusion loop antibodies have been proposed to function as cross-protective antibodies within the pool of heterotypic neutralizing antibodies [35]. High frequencies of circulating DENV-specific T lymphocyte responses have also been proposed to provide some measure of protective immunity [36], [37]. Whatever measure is used will need to be validated with well characterized samples from studies with clinically relevant outcomes. In our study, it is interesting that PRNT using homologous DENV-2 strains did not strengthen the association with protection as compared with PRNT using DENV-2 reference strain. Furthermore, using an altogether different DENV-2 genotype (e.g., Asian-American) from the infecting Asian I genotype did not weaken the association. These results suggest that strain differences in PRNT may be of little importance in assessing protection. Other more functionally relevant assays may still be able to detect clinically relevant strain-related differences.

Although the samples in our study were unique and informative, the number that met testing criteria was small, limiting the power of our analysis. Nevertheless, the fact that significant associations were found despite these small numbers supports the strength of the relationships. Larger sample numbers may have revealed more subtle associations, for example, between different heterotypic NT combinations and protection from infection. Because of the small numbers, we used data from two different geographic cluster studies over multiple years. The inclusion of both adults and children with likely differing immunological backgrounds may have affected our findings for the DENV-2 analysis, possibly accounting for the higher DENV-2 NTs associated with protection. We also found that “susceptible” subjects were more likely to be DENV NT naïve than “non-susceptible” subjects. Thus, part of the increased likelihood of infection associated with lower baseline NTs may have been due to susceptibility to primary versus secondary infections. In addition, as mentioned earlier, we could not be certain that “non-susceptible” subjects had, in fact, been exposed to DENV infection. If, however, some of the “non-susceptible” subjects had simply been unexposed rather than protected from infection, the likelihood of detecting an association between NT and protection would have been even less. Finally, we were not able to characterize the clinical status of infected subjects because no follow up visits took place after day 15. Therefore, we were unable to make conclusions about associations with clinical severity which may be a more relevant endpoint for vaccine evaluation than detection of viremia by PCR.

In our study population, neutralizing antibody titers were associated with protection against DENV-1, -2 and -4. NT levels required for this protection varied for these three serotypes, but were likely affected by the preceding epidemiological and immunological history of the subjects. These findings will help inform ongoing studies of dengue epidemiology and the development of dengue vaccine candidates.

## Supporting Information

Figure S1 **Pre-exposure neutralizing antibody titers (NTs) in dengue virus serotype 1 (DENV-1) “susceptible” and “non-susceptible subjects.** (a) six DENV-1 PCR positive (“susceptible”) subjects; (b) 13 DENV-1 PCR negative (“non-susceptible”) subjects. NTs for each subject are against the reference strains for DENV-1, -2, -3, -4 and Japanese encephalitis virus (JEV) (red, blue, green, purple and orange, respectively).
(TIFF)

Figure S2 **Pre-exposure neutralizing antibody titers (NTs) in dengue virus serotype 4 (DENV-4) “susceptible” and “non-susceptible” subjects.** (a) six DENV-4 PCR positive (“susceptible”) subjects; (b) nine DENV-4 PCR negative (“non-susceptible”) subjects. NTs for each subject are against the reference strains for DENV-1, -2, -3, -4 and Japanese encephalitis virus (JEV) (red, blue, green, purple and orange, respectively).
(TIFF)

Figure S3 **Pre-exposure neutralizing antibody titers (NTs) in dengue virus serotype 2 (DENV-2) “susceptible” and “non-susceptible” subjects.** Each row shows one PCR positive (“susceptible”) subject and two matched PCR negative (“non-susceptible”) subjects. NTs for each subject are against (in order from left to right for each subject) the reference strains for DENV-1, -2, -3, -4 and Japanese encephalitis virus (JEV), and six different DENV-2 strains (Asian-American 1982 [#D82-165], Asian I 1974 [#D74-066], Asian I 1984 [#D84-501], Asian I 1994 [#D94-035], Asian I 2004 [#KDS00305]), and homologous DENV-2 Asian I strains).
(TIFF)

Figure S4 **Receiver operating characteristic (ROC) curves for homotypic neutralizing antibody titers (NTs) against reference strains.** Curves discriminate between dengue virus (DENV) PCR positive and negative subjects for: (a) all serotypes combined; (b) DENV-1; (c) DENV-4; (d) DENV-2.
(TIFF)

Table S1 **Characteristics of dengue PCR positive (“susceptible”) and negative (“non-susceptible”) subjects.**
(TIFF)

Table S2 **Logistic regression analysis of the relationship between homotypic neutralizing antibody titers and dengue PCR status.^a^**
(TIFF)

Table S3 **Comparison of logistic regression models of neutralizing antibody titers to predict dengue PCR status. Models using neutralizing antibody titer alone and adjusted for age.**
(TIFF)

Table S4 **Contingency tables showing relationship between homotypic neutralizing antibody titer cutoff values and dengue PCR status.**
(TIFF)

Checklist S1 **STROBE checklist.**
(PDF)

## References

[1] Gubler DJ (2002) The global emergence/resurgence of arboviral diseases as public health problems. Arch Med Res 33: 330–342. 12234522 10.1016/s0188-4409(02)00378-8

[2] Lindenbach BD, Rice CM (2003) Molecular biology of flaviviruses. Adv Virus Res 59: 23–61. 14696326 10.1016/s0065-3527(03)59002-9

[3] Bhatt S, Gething PW, Brady OJ, Messina JP, Farlow AW, et al (2013) The global distribution and burden of dengue. Nature 496: 504–507. 23563266 10.1038/nature12060 PMC3651993

[4] Sabchareon A, Wallace D, Sirivichayakul C, Limkittikul K, Chanthavanich P, et al (2012) Protective efficacy of the recombinant, live-attenuated, CYD tetravalent dengue vaccine in Thai schoolchildren: a randomised, controlled phase 2b trial. Lancet 380: 1559–1567. 22975340 10.1016/S0140-6736(12)61428-7

[5] Halstead SB (1988) Pathogenesis of dengue: challenges to molecular biology. Science 239: 476–481. 3277268 10.1126/science.3277268

[6] Halstead SB (2003) Neutralization and antibody-dependent enhancement of dengue viruses. Adv Virus Res 60: 421–467. 14689700 10.1016/s0065-3527(03)60011-4

[7] Sabin AB (1952) Research on dengue during World War II. Am J Trop Med Hyg 1: 30–50. 14903434 10.4269/ajtmh.1952.1.30

[8] Wahala WM, Silva AM (2011) The human antibody response to dengue virus infection. Viruses 3: 2374–2395. 22355444 10.3390/v3122374 PMC3280510

[9] Reich NG, Shrestha S, King AA, Rohani P, Lessler J, et al (2013) Interactions between serotypes of dengue highlight epidemiological impact of cross-immunity. J R Soc Interface 10: 20130414. 23825116 10.1098/rsif.2013.0414 PMC3730691

[10] Anderson KB, Gibbons RV, Cummings DA, Nisalak A, Green S, et al (2014) A shorter time interval between first and second dengue infections is associated with protection from clinical illness in a school-based cohort in Thailand. J Infect Dis 209: 360–368. 23964110 10.1093/infdis/jit436 PMC3883164

[11] Montoya M, Gresh L, Mercado JC, Williams KL, Vargas MJ, et al (2013) Symptomatic versus inapparent outcome in repeat dengue virus infections is influenced by the time interval between infections and study year. PLoS Negl Trop Dis 7: e2357. 23951377 10.1371/journal.pntd.0002357 PMC3738476

[12] Guzman MG, Alvarez M, Rodriguez-Roche R, Bernardo L, Montes T, et al (2007) Neutralizing antibodies after infection with dengue 1 virus. Emerg Infect Dis 13: 282–286. 17479892 10.3201/eid1302.060539 PMC2725871

[13] Kliks SC, Nisalak A, Brandt WE, Wahl L, Burke DS (1989) Antibody-dependent enhancement of dengue virus growth in human monocytes as a risk factor for dengue hemorrhagic fever. Am J Trop Med Hyg 40: 444–451. 2712199 10.4269/ajtmh.1989.40.444

[14] Endy TP, Nisalak A, Chunsuttitwat S, Vaughn DW, Green S, et al (2004) Relationship of Preexisting Dengue Virus (DV) Neutralizing Antibody Levels to Viremia and Severity of Disease in a Prospective Cohort Study of DV Infection in Thailand. J Infect Dis 189: 990–1000. 14999601 10.1086/382280

[15] Kochel TJ, Watts DM, Halstead SB, Hayes CG, Espinoza A, et al (2002) Effect of dengue-1 antibodies on American dengue-2 viral infection and dengue haemorrhagic fever. Lancet 360: 310–312. 12147378 10.1016/S0140-6736(02)09522-3

[16] Sirivichayakul C, Sabchareon A, Limkittikul K, Yoksan S (2014) Plaque reduction neutralization antibody test does not accurately predict protection against dengue infection in Ratchaburi cohort, Thailand. Virol J 11: 48. 24620925 10.1186/1743-422X-11-48 PMC3975240

[17] Rainwater-Lovett K, Rodriguez-Barraquer I, Cummings DA, Lessler J (2012) Variation in dengue virus plaque reduction neutralization testing: systematic review and pooled analysis. BMC Infect Dis 12: 233. 23020074 10.1186/1471-2334-12-233 PMC3519720

[18] Yoon IK, Getis A, Aldstadt J, Rothman AL, Tannitisupawong D, et al (2012) Fine scale spatiotemporal clustering of dengue virus transmission in children and Aedes aegypti in rural Thai villages. PLoS Negl Trop Dis 6: e1730. 22816001 10.1371/journal.pntd.0001730 PMC3398976

[19] Yoon IK, Rothman AL, Tannitisupawong D, Srikiatkhachorn A, Jarman RG, et al (2012) Underrecognized mildly symptomatic viremic dengue virus infections in rural Thai schools and villages. J Infect Dis 206: 389–398. 22615312 10.1093/infdis/jis357 PMC3490697

[20] Mammen MP, Pimgate C, Koenraadt CJ, Rothman AL, Aldstadt J, et al (2008) Spatial and temporal clustering of dengue virus transmission in Thai villages. PLoS Med 5: e205. 18986209 10.1371/journal.pmed.0050205 PMC2577695

[21] Klungthong C, Gibbons RV, Thaisomboonsuk B, Nisalak A, Kalayanarooj S, et al (2007) Dengue Viral Detection using Whole Blood for RT-PCR and Viral Isolation. J Clin Microbiol 10.1128/JCM.00305-07 PMC1951229 17522268

[22] Lanciotti RS, Calisher CH, Gubler DJ, Chang GJ, Vorndam AV (1992) Rapid detection and typing of dengue viruses from clinical samples by using reverse transcriptase-polymerase chain reaction. J Clin Microbiol 30: 545–551. 1372617 10.1128/jcm.30.3.545-551.1992 PMC265106

[23] Innis BL, Nisalak A, Nimmannitya S, Kusalerdchariya S, Chongswasdi V, et al (1989) An enzyme-linked immunosorbent assay to characterize dengue infections where dengue and Japanese encephalitis co-circulate. Am J Trop Med Hyg 40: 418–427. 2540664 10.4269/ajtmh.1989.40.418

[24] Russell PK, Nisalak A, Sukhavachana P, Vivona S (1967) A plaque reduction test for dengue virus neutralization antibodies. J Immunol 99: 285–290. 6031202

[25] Thomas SJ, Nisalak A, Anderson KB, Libraty DH, Kalayanarooj S, et al (2009) Dengue plaque reduction neutralization test (PRNT) in primary and secondary dengue virus infections: How alterations in assay conditions impact performance. Am J Trop Med Hyg 81: 825–833. 19861618 10.4269/ajtmh.2009.08-0625 PMC2835862

[26] Agresti A (2002) Categorical Data Analysis; Balding DJ, Cressie NAC, Fitzmaurice GM, Johnstone IM, Molenberghs G, et al.., editors. Hoboken, NJ: John Wiley and Sons, Inc.

[27] Pepe M, Longton G, Janes H (2009) Estimation and Comparison of Receiver Operating Characteristic Curves. Stata J 9: 1. 20161343 PMC2774909

[28] Carpenter J, Bithell J (2000) Bootstrap confidence intervals: when, which, what? A practical guide for medical statisticians. Stat Med 19: 1141–1164. 10797513 10.1002/(sici)1097-0258(20000515)19:9<1141::aid-sim479>3.0.co;2-f

[29] Robin X, Turck N, Hainard A, Tiberti N, Lisacek F, et al (2011) pROC: an open-source package for R and S+ to analyze and compare ROC curves. BMC Bioinformatics 12: 77. 21414208 10.1186/1471-2105-12-77 PMC3068975

[30] Yoon IK, Srikiatkhachorn A, Hermann L, Buddhari D, Scott TW, et al (2013) Characteristics of mild dengue virus infection in Thai children. Am J Trop Med Hyg 89: 1081–1087. 24127167 10.4269/ajtmh.13-0424 PMC3854884

[31] Chawla T, Chan KR, Zhang SL, Tan HC, Lim AP, et al (2013) Dengue virus neutralization in cells expressing Fc gamma receptors. PLoS One 8: e65231. 23717696 10.1371/journal.pone.0065231 PMC3661447

[32] Moi ML, Lim CK, Chua KB, Takasaki T, Kurane I (2012) Dengue virus infection-enhancing activity in serum samples with neutralizing activity as determined by using FcgammaR-expressing cells. PLoS Negl Trop Dis 6: e1536. 22389741 10.1371/journal.pntd.0001536 PMC3289619

[33] de Alwis R, Smith SA, Olivarez NP, Messer WB, Huynh JP, et al (2012) Identification of human neutralizing antibodies that bind to complex epitopes on dengue virions. Proc Natl Acad Sci U S A 109: 7439–7444. 22499787 10.1073/pnas.1200566109 PMC3358852

[34] Messer WB, de Alwis R, Yount BL, Royal SR, Huynh JP, et al (2014) Dengue virus envelope protein domain I/II hinge determines long-lived serotype-specific dengue immunity. Proc Natl Acad Sci U S A 111: 1939–1944. 24385585 10.1073/pnas.1317350111 PMC3918811

[35] Lai CY, Williams KL, Wu YC, Knight S, Balmaseda A, et al (2013) Analysis of cross-reactive antibodies recognizing the fusion loop of envelope protein and correlation with neutralizing antibody titers in Nicaraguan dengue cases. PLoS Negl Trop Dis 7: e2451. 24069496 10.1371/journal.pntd.0002451 PMC3777924

[36] Hatch S, Endy TP, Thomas S, Mathew A, Potts J, et al (2011) Intracellular cytokine production by dengue virus-specific T cells correlates with subclinical secondary infection. J Infect Dis 203: 1282–1291. 21335561 10.1093/infdis/jir012 PMC3069729

[37] Weiskopf D, Angelo MA, de Azeredo EL, Sidney J, Greenbaum JA, et al (2013) Comprehensive analysis of dengue virus-specific responses supports an HLA-linked protective role for CD8+ T cells. Proc Natl Acad Sci U S A 110: E2046–2053. 23580623 10.1073/pnas.1305227110 PMC3670335

