# Supplementary figures and images for: Dengue Virus Neutralizing Antibody Levels Associated with Protection from Infection in Thai Cluster Studies
Source: PLoS Negl Trop Dis. 2014 Oct 16;8(10):e3230. doi: 10.1371/journal.pntd.0003230 (PMC4199527; doi:10.1371/journal.pntd.0003230)

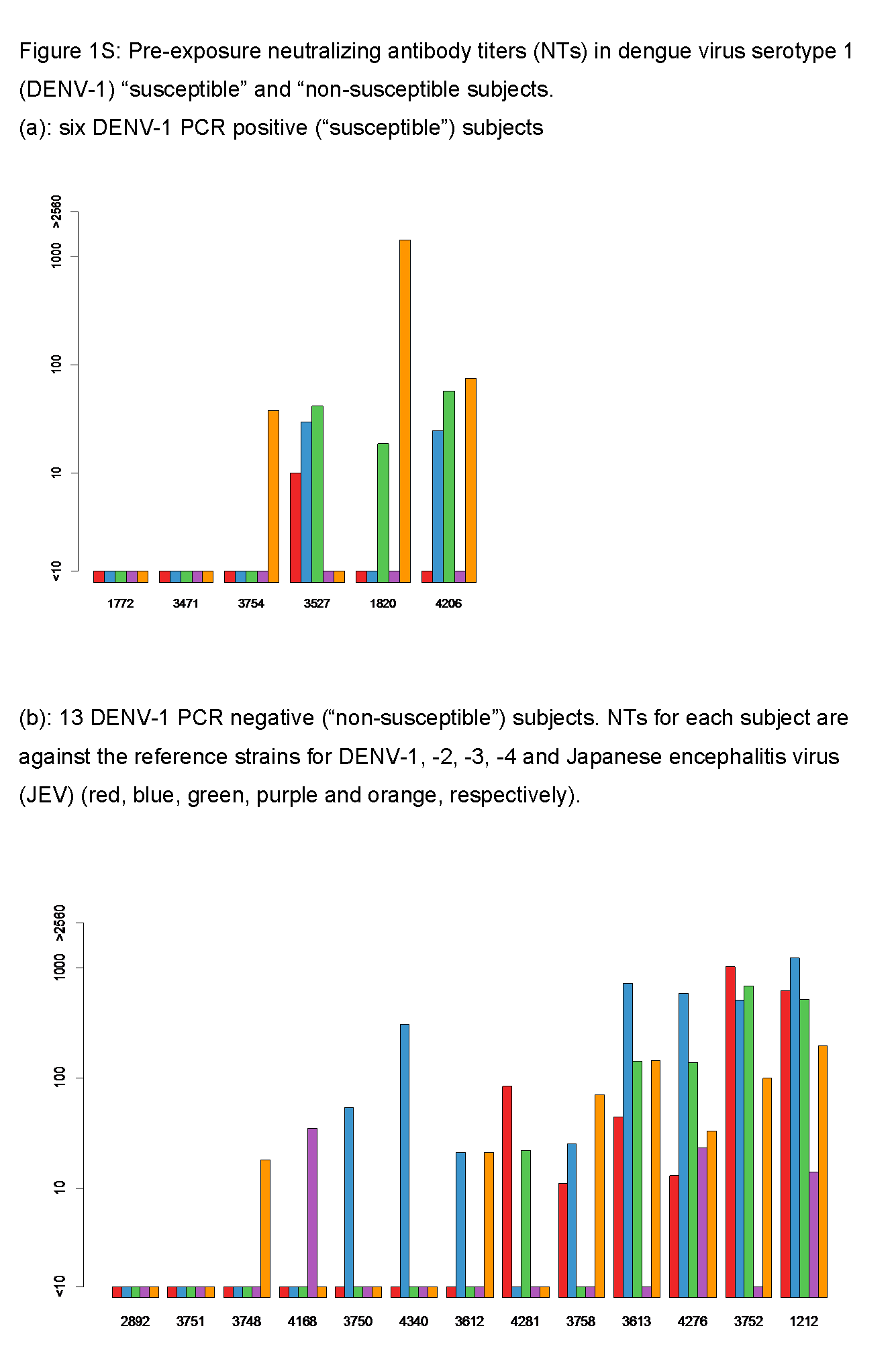

Supplement: Figure S1 — Pre-exposure neutralizing antibody titers (NTs) in dengue virus serotype 1 (DENV-1) “susceptible” and “non-susceptible subjects. (a) six DENV-1 PCR positive (“susceptible”) subjects; (b) 13 DENV-1 PCR negative (“non-susceptible”) subjects. NTs for each subject are against the reference strains for DENV-1, -2, -3, -4 and Japanese encephalitis virus (JEV) (red, blue, green, purple and orange, respectively). (TIFF) [file pntd.0003230.s001.tiff]

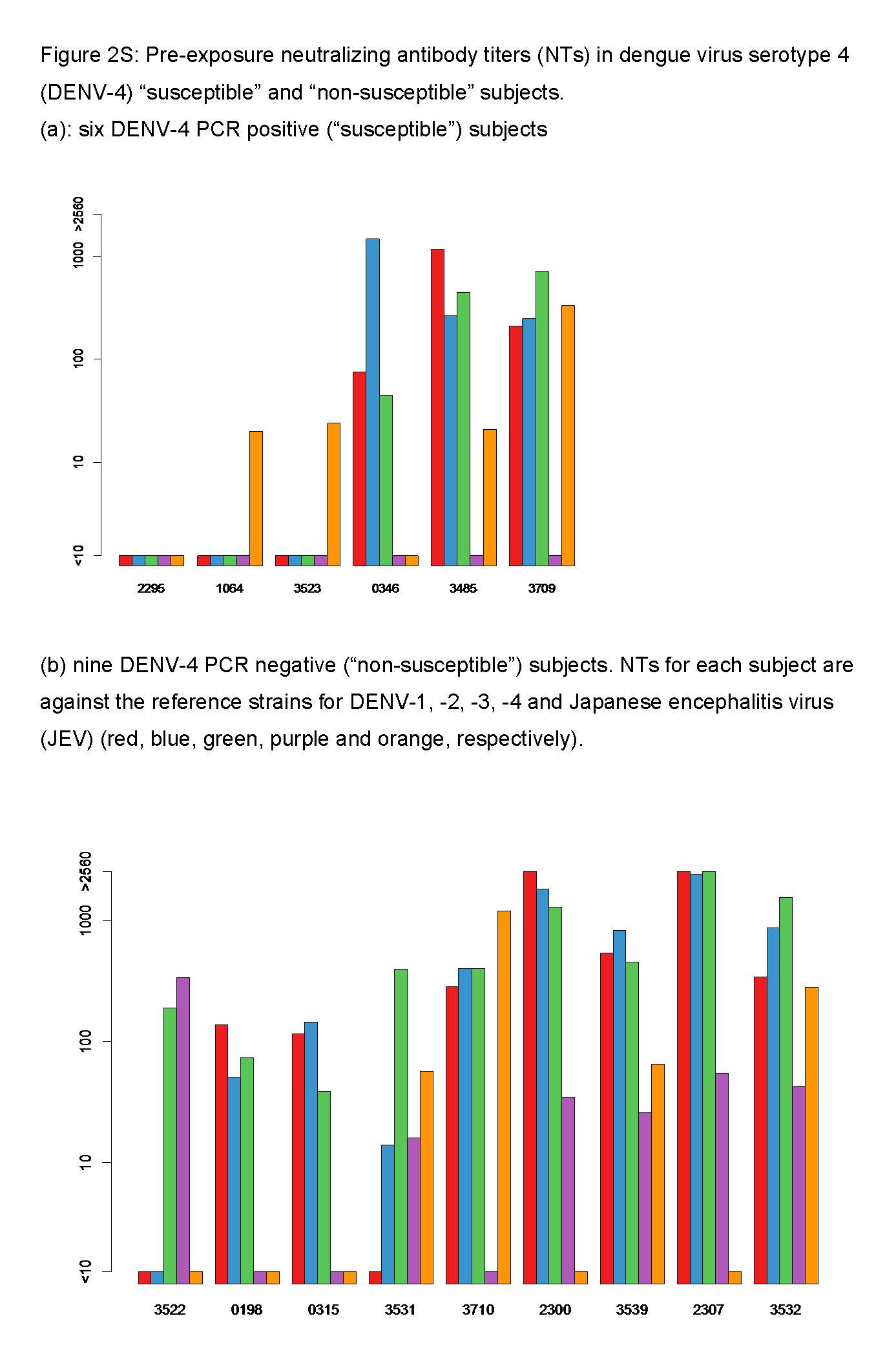

Supplement: Figure S2 — Pre-exposure neutralizing antibody titers (NTs) in dengue virus serotype 4 (DENV-4) “susceptible” and “non-susceptible” subjects. (a) six DENV-4 PCR positive (“susceptible”) subjects; (b) nine DENV-4 PCR negative (“non-susceptible”) subjects. NTs for each subject are against the reference strains for DENV-1, -2, -3, -4 and Japanese encephalitis virus (JEV) (red, blue, green, purple and orange, respectively). (TIFF) [file pntd.0003230.s002.tiff]

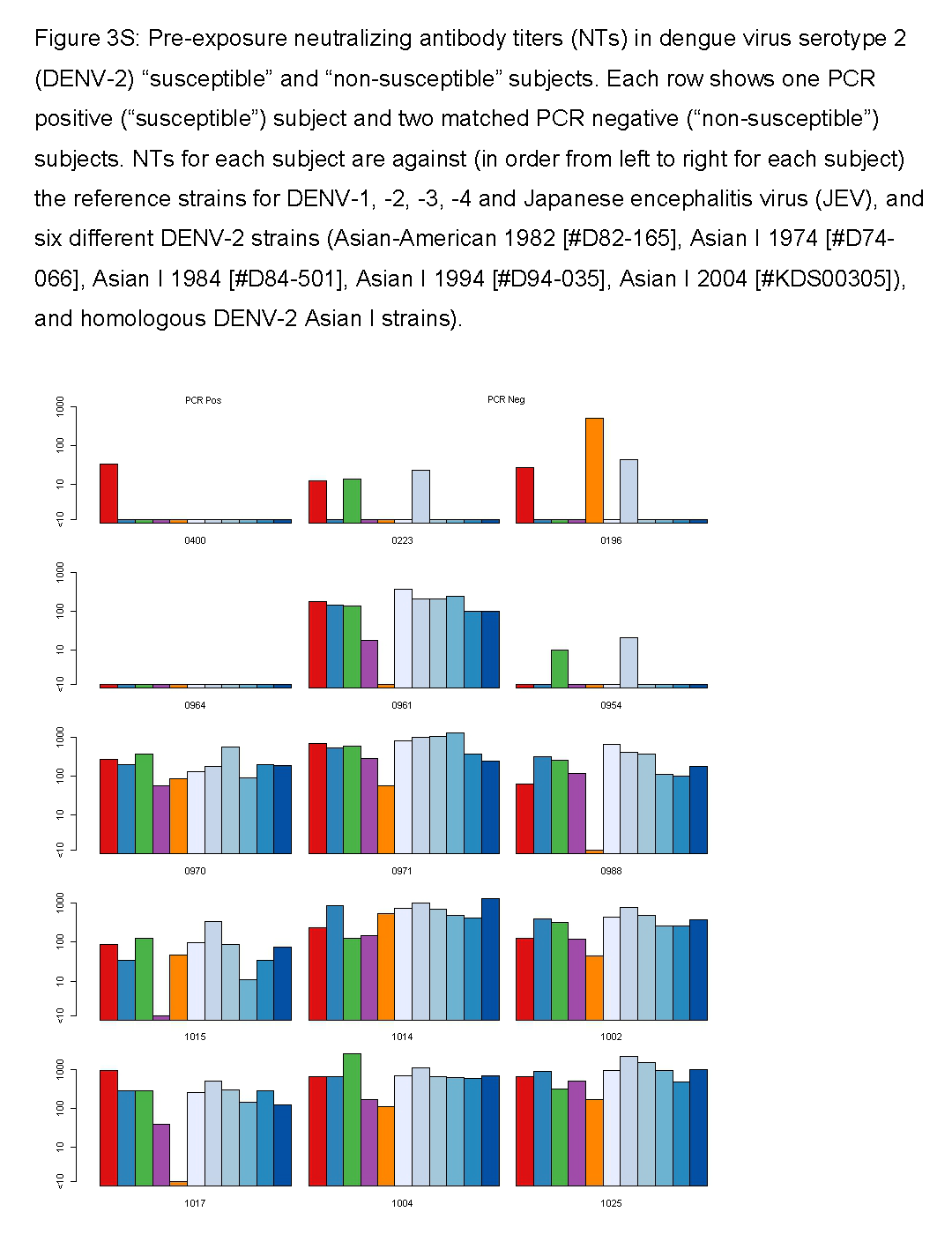

Supplement: Figure S3 — Pre-exposure neutralizing antibody titers (NTs) in dengue virus serotype 2 (DENV-2) “susceptible” and “non-susceptible” subjects. Each row shows one PCR positive (“susceptible”) subject and two matched PCR negative (“non-susceptible”) subjects. NTs for each subject are against (in order from left to right for each subject) the reference strains for DENV-1, -2, -3, -4 and Japanese encephalitis virus (JEV), and six different DENV-2 strains (Asian-American 1982 [#D82-165], Asian I 1974 [#D74-066], Asian I 1984 [#D84-501], Asian I 1994 [#D94-035], Asian I 2004 [#KDS00305]), and homologous DENV-2 Asian I strains). (TIFF) [file pntd.0003230.s003.tiff]

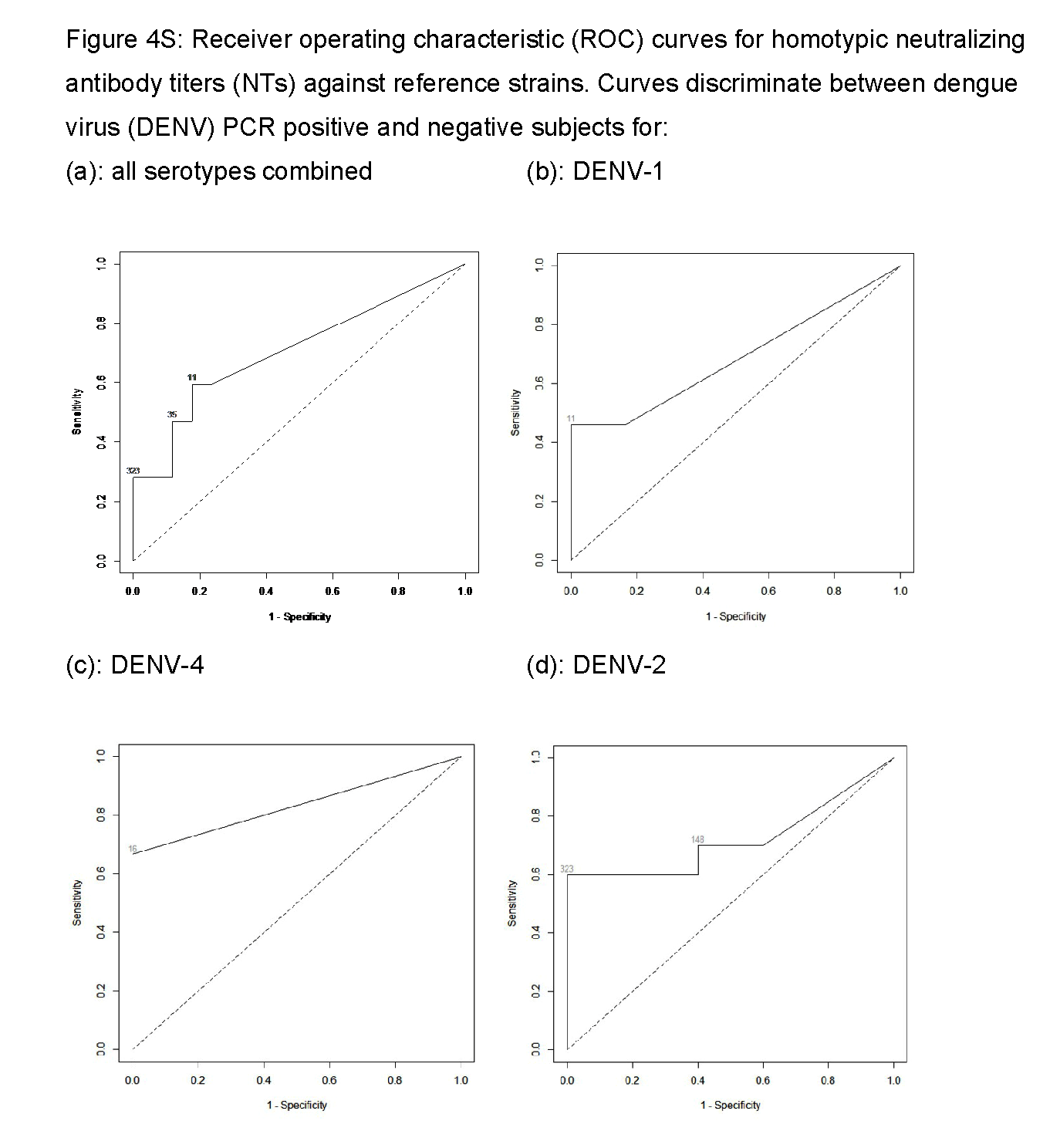

Supplement: Figure S4 — Receiver operating characteristic (ROC) curves for homotypic neutralizing antibody titers (NTs) against reference strains. Curves discriminate between dengue virus (DENV) PCR positive and negative subjects for: (a) all serotypes combined; (b) DENV-1; (c) DENV-4; (d) DENV-2. (TIFF) [file pntd.0003230.s004.tiff]

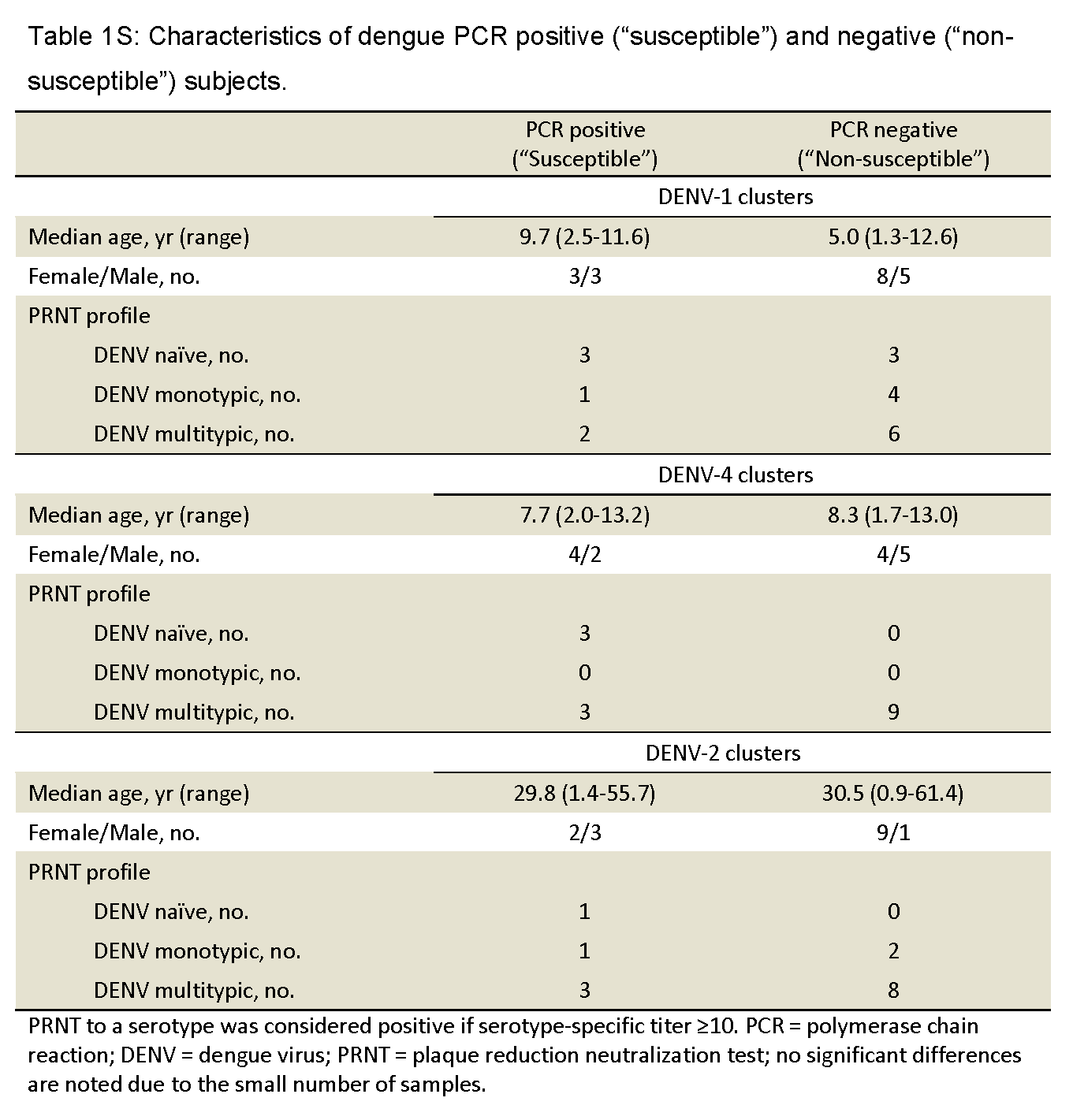

Supplement: Table S1 — Characteristics of dengue PCR positive (“susceptible”) and negative (“non-susceptible”) subjects. (TIFF) [file pntd.0003230.s005.tiff]

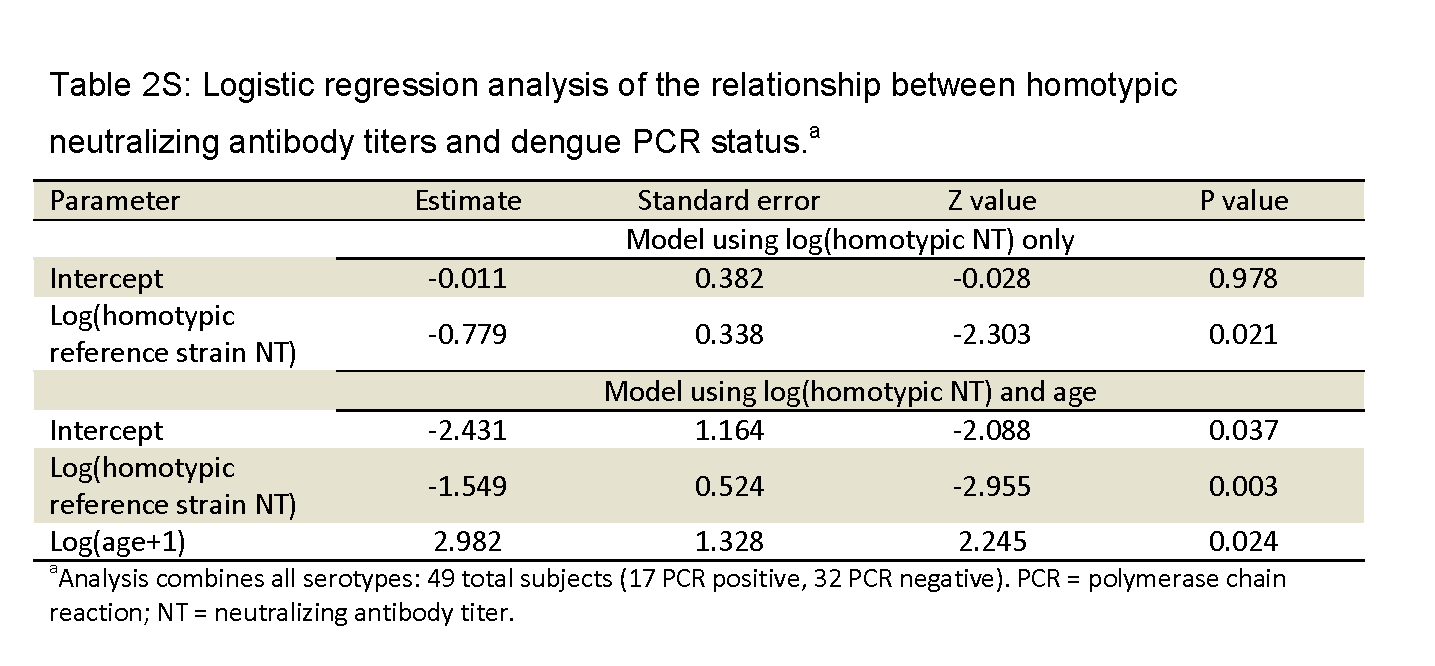

Supplement: Table S2 — Logistic regression analysis of the relationship between homotypic neutralizing antibody titers and dengue PCR status.a (TIFF) [file pntd.0003230.s006.tiff]

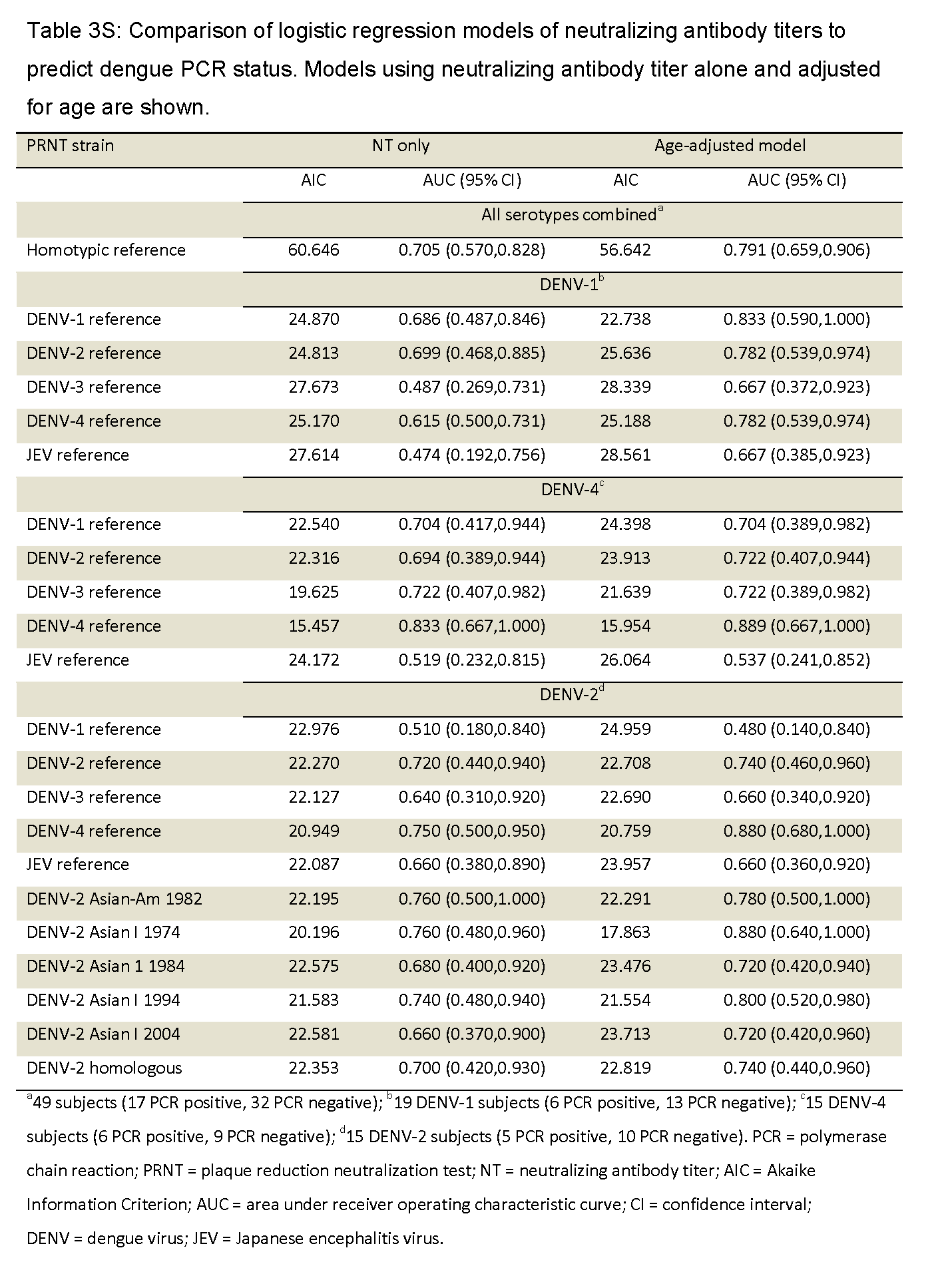

Supplement: Table S3 — Comparison of logistic regression models of neutralizing antibody titers to predict dengue PCR status. Models using neutralizing antibody titer alone and adjusted for age. (TIFF) [file pntd.0003230.s007.tiff]

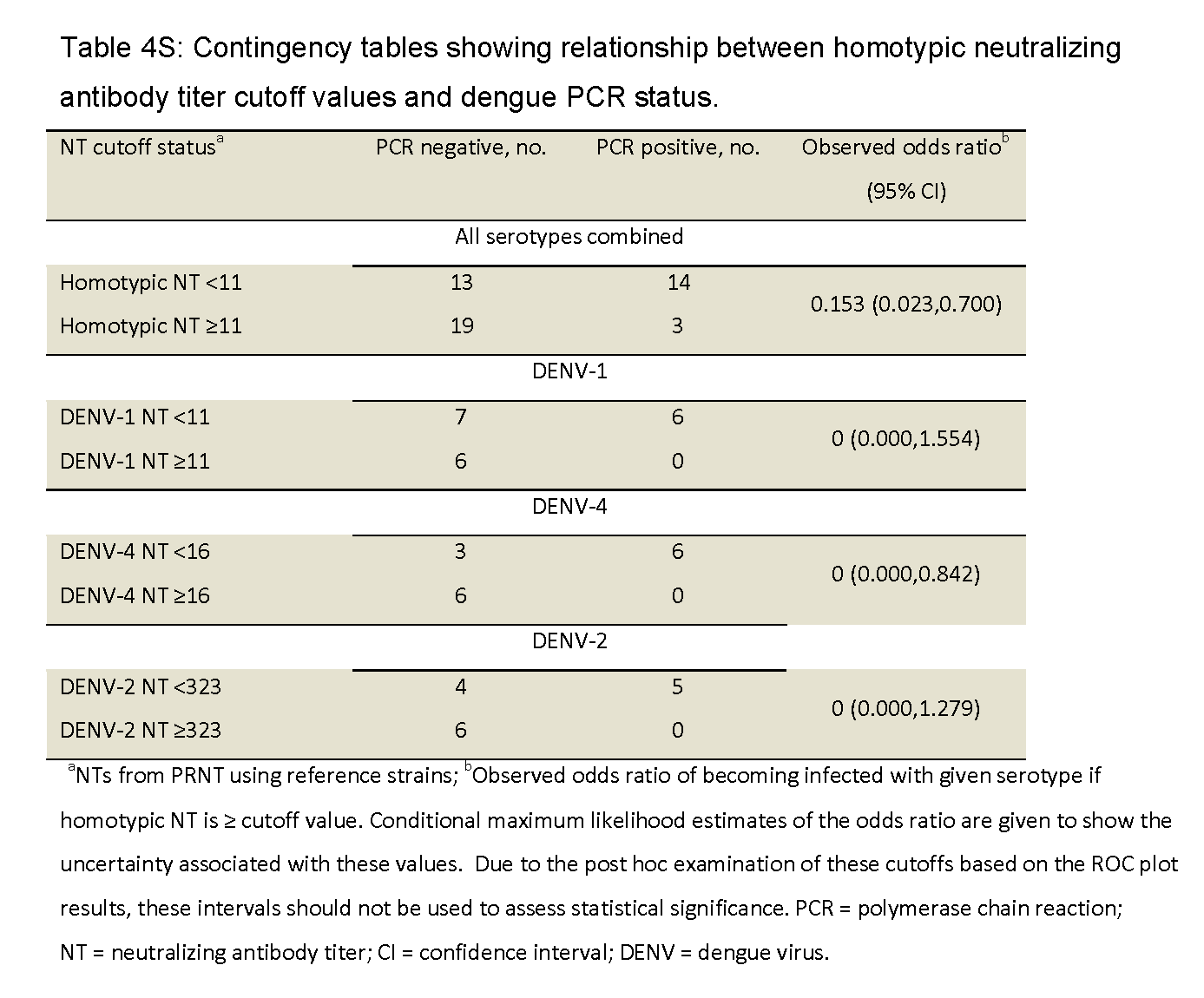

Supplement: Table S4 — Contingency tables showing relationship between homotypic neutralizing antibody titer cutoff values and dengue PCR status. (TIFF) [file pntd.0003230.s008.tiff]
